# Catarrh and Dyspepsia

**Published:** 1881-07-20

**Authors:** A. P. Whitehead


### CATARRH AND DYSPEPSIA.

BY A. P. WHITEHEAD, M. D.

The relation of dyspepsia to catarrh is of such importance in connection with the treatment of the latter disease, that it is a matter for wonder that it has not been more prominently brought to notice in the many and lengthy treatises upon the subject. That dyspepsia is a frequent accompaniment, and often precedes a chronic naso-pharyngeal catarrh, is no new statement; but that it invariably exists and generally long ante-dates a chronic catarrh, and is the forerunner, and, to a certain extent, the cause of the presence of catarrh. is the opinion of the writer, confirmed by oft-repeated observation in the treatment of that stubborn affection designated chronic catarrh.

The treatment recommended by writers upon the subject has been confined too much to the seat of disease, with a view to bringing about a modification of the affected mucous membrane by the application of astringents or caustics in a more or less dilute or concentrated form, and even to the removal of portions of the lining membrane of the nose by mechanical means. The diversity in the methods adoptdd have been confined to a simple difference in the destructive character of the applications used—chromic and nitric acid, the actual cautery and the serrated forceps capping the climax. Safer and more rational means of treatment have been too much ignored; the treatment of the dyspepsia, *having in view the relief of the catarrh*, has been almost overlooked.

In the mind of a patient suffering from catarrh there can be no possible connection with it and dyspepsia; and hence it is not an uncommon occurrence for him to be undergoing treatment at the hands of a specialist for the catarrhal trouble while being treated by the family physician for dyspepsia.

The usual form of dyspepsia accompanying catarrh is characterized by a somewhat enlarged tongue, bearing upon its sides the impressions of the bicuspids and first molar teeth; in color, pale or slightly bluish, the latter, especially, after a meal; near the tip smooth, dotted here and there with small, bright, pink or red slightly raised follicles; at the base, a slight whitish fur, and a more or less deep fissure in the center, extending half-way to the tip. This is clearly the tongue of chronic, atonic dyspepsia, with more or less enlargement of the stomach and permanent thickening of its mucous lining, not unlike the thickening of the nasal and pharyngeal mucous membrane, with which it is directly continuous.

The so-called "*hygiene of catarrh*" is the hygiene of dyspepsia. The greatest benefit derived from a hygienic habit is more directly owing to the improvement of the impaired functions of the stomach than to any direct benefit to the nose and pharynx; for, above all other affections, dyspepsia demands a rigid observance of the laws of hygiene for its treatment.—*Western Lancet.*

